# Gene flow between diploid and tetraploid junipers - two contrasting evolutionary pathways in two *Juniperus* populations

**DOI:** 10.1186/s12862-020-01688-3

**Published:** 2020-11-09

**Authors:** Perla Farhat, Sonja Siljak-Yakovlev, Nicolas Valentin, Carlos Fabregat, Silvia Lopez-Udias, Carlos Salazar-Mendias, Joaquín Altarejos, Robert P. Adams

**Affiliations:** 1 Biology Department, Baylor University, Waco, TX 76798 USA; 2 Present address: Key Laboratory of Bio-resources and Eco-environment, College of Life Sciences, Sichuan University, Chengdu, Sichuan 610064 China; 3 Université Paris-Saclay, CNRS, AgroParisTech, Ecologie Systématique Evolution, 91405 Orsay, France; 4 Institute of Integrative Biology of the Cell (I2BC), CEA, CNRS, Université Paris-Saclay, 91198 Gif-sur-Yvette, France; 5 Jardí Botànic de la Universitat de València, 46008 València, Spain; 6 Departamento de Biología Animal, Biología Vegetal y Ecología. Universidad de Jaén, 23071 Jaén, Spain; 7 Departamento de Química Inorgánica y Orgánica, Universidad de Jaén, 23071 Jaén, Spain

**Keywords:** *Juniperus*, Gene flow, Polyploidy, Triploid bridge, Hybridization, Introgression, Spain, Conifer evolution

## Abstract

**Background:**

Gene flow and polyploidy have been found to be important in *Juniperus* evolution. However, little evidence has been published elucidating the association of both phenomena in juniper taxa in the wild. Two main areas were studied in Spain (Eastern Iberian Range and Sierra de Baza) with both diploid and tetraploid taxa present in sympatry. Gene flow and ploidy level were assessed for these taxa and the resulted offspring.

**Results:**

Twenty-two allo-triploid hybrids between *J. sabina* var. *sabina* and *J. thurifera* were found in the Eastern Iberian Range population. However, in the Sierra de Baza population no triploids were found. Instead, 18 allo-tetraploid hybrids between two tetraploid taxa: *J. sabina* var. *balkanensis* and *J. thurifera* were discovered. High genetic diversity was exhibited among the tetraploid hybrids at Sierra de Baza, in contrast to the genetically identical triploid hybrids at the Eastern Iberian Range; this suggests meiotic difficulties within the triploid hybrids. In addition, unidirectional gene flow was observed in both studied areas.

**Conclusion:**

Polyploidy and hybridization can be complementary partners in the evolution of *Juniperus* taxa in sympatric occurrences. *Juniperus* was shown to be an ideal coniferous model to study these two phenomena, independently or in concert.

## Background

Hybridization and polyploidy have been found to be widespread among plant groups, shaping their evolution and adaptation [1, 2]. The conifers, however, seem to differ from angiosperms in that hybridization has been found to be more common than polyploidy, which has been estimated to be very rare at approximately 1.5% [3, 4]. Recently, the *Juniperus* L*.*, a monophyletic Cupressaceae genus, [5, 6] was shown to have an exceptional high rate of polyploidy compared to all other conifers [7]. *Juniperus* is the most diverse genus within the Cupressaceae and the second within conifers. It contains 75 species and 40 varieties in 3 monophyletic sections *Caryocedrus*, *Juniperus* and *Sabina* [6]. A recent study of this genus discovered 15% of the taxa are tetraploid (2*n* = 4*x* = 44) and one taxon, *Juniperus foetidissima* Willd., is a hexaploid (2*n* = 6*x* = 66) [7]. Thus, polyploidy has been shown to be highly implicated in *Juniperus* evolution. In addition, hybridization has been found to be an important phenomenon in *Juniperus* with many cases of hybridization reported including: *J. arizonica* (R. P. Adams) R. P. Adams x *J. coahuilensis* (Mart.) Gaussen ex R. P. Adams [8], *J. maritima* R. P. Adams x *J. scopulorum* Sarg. [9, 10], *J. virginiana* var. *silicicola* (Small) E. Murray x *J. bermudiana* L. [11], *J. virginiana* L. x *J. horizontalis* Moench [12] and *J. osteosperma* (Torr.) Little x *J. occidentalis* Hook. [13, 14]. The evolutionary impact of hybridization coupled with polyploidy through allopolyploidy has not been very well elaborated to date in *Juniperus*.

However, recently, a potential allo-tetraploid taxon, *Juniperus sabina* var. *balkanensis* R. P. Adams and A. N. Tashev has been discovered [15, 16]. This variety was described principally based on the DNA sequence differences with only a few morphological differences with its sister variety, *J. sabina* var. *sabina* [15]*.* The newly described variety was shown to have participated in a chloroplast capture event from *J. thurifera* L., following an ancient hybridization between the tetraploid *J. thurifera* and the diploid *J. sabina* [15]. All populations of *J. sabina* var. *balkanensis,* analyzed at present, have been found to be tetraploid. In contrast, all samples of *J. sabina* var. *sabina* have been found to be diploid [16]. The current known geographical distribution does not overlap, even though the distribution of *J. sabina* var. *sabina* is widespread from Spain into China. Currently, *J. sabina* var. *balkanensis* has been confirmed by DNA analyses from Italy, the Balkans and the western part of Turkey [17]. Despite the difference in ploidy level between *J. thurifera* (2*n* = 4*x*) and *J. sabina* var. *sabina* (2*n* = 2*x*), first evidence of allo-triploid hybrids between those two taxa have been recently discovered in the French Alps, where the taxa occur in sympatry [18]. However, in Spain, *J. sabina* var. *sabina* and *J. thurifera* are occasionally sympatric and a putative hybrid has been described as *Juniperus x cerropastorensis* J.M. Aparicio & P.M. Uribe-Echebarria, based on their intermediate morphology [19].

**Fig. 1:**
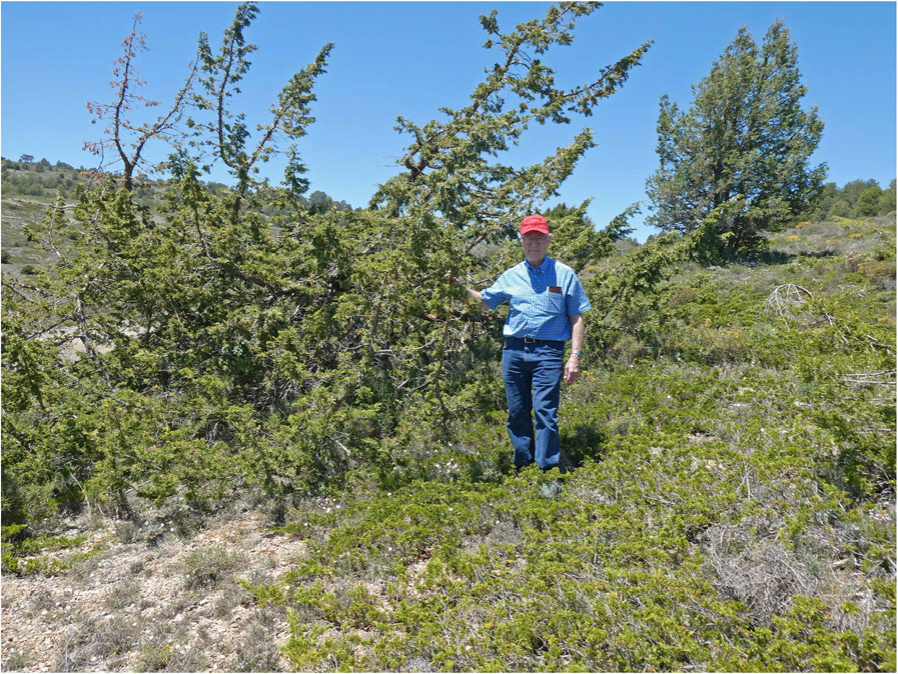
*Juniperus* studied taxa from Eastern Iberian Range. *Juniperus sabina* (prostrate, lower right), *J. thurifera* (tree, upper right) and hybrid *Adams 15655* (shrub, center-left) with RPA. Photo by CF.

The hybrid has an irregular, shrubby shape (Fig. 1), not erect as *J. thurifera*, nor as prostrate as *J. sabina*. Those potential hybrids differ by their shape and branches, female cone size, and the number of seeds per female cone. *Juniperus x cerropastorensis* has been reported in a small area in Spain: in three counties; Castellón, Teruel and Valencia in the Eastern Iberian Range [19].

Because no molecular evidence has been reported on these putative hybrid plants, one objective of this study was to confirm the hybridization between *J. sabina* and *J. thurifera* based on nuclear and chloroplast markers in areas of geographical sympatry in Spain. Because the putative parents (*J. sabina* and *J. thurifera*) are respectively diploid and tetraploid, ploidy of the hybrid(s) is of considerable interest. Therefore, the ploidy levels of the samples were determined by flow cytometry. The extreme rarity of inter-ploidy interspecific hybridization in conifers in the wild makes studying these juniper putative hybrids of high importance especially for discovering potential pathways of evolution in this genus.

In addition, the only locality found to date, where the tetraploid *J. sabina* var. *balkanensis* and the diploid *J. sabina* var. *sabina* grow in sympatry is at Sierra de Baza, Granada province, Spain. Thus, our second aim was to study this unusual opportunity to find out if these two varieties interact together via gene flow and to measure the resulting ploidy level of hybrids (if any). This rare case of sympatry is a significant event that may give insight into the reproductive evolution of varieties (or taxa) with different ploidy levels within the same species of juniper.

## Results

### Genome size of parents and putative hybrids

Genome size (GS) was successfully assessed for all individuals sampled from the Eastern Iberian Range (hereafter referred to as E Iberian Range) and Sierra de Baza areas.

In the E Iberian Range, *J. thurifera* showed a genome size ranging from 41.79 pg to 44.84 pg with a mean of 42.55 pg (σ =0.86 pg). *Juniperus sabina* sampled from this population hold a genome size of 20.79 pg to 22.51 pg with a mean of 21.83 pg (σ =0.41 pg).

The 22 *J. x cerropastorensis* samples (putative hybrids based on field observations) showed a genome size from 31.63 pg to 35.22 pg with a mean GS of 33.24 pg (σ =0.83 pg).

In the Sierra de Baza, thirty samples of *J. sabina* (*J. sabina* var. *sabina* and *J. sabina* var. *balkanensis*) were studied where two groups of GS were found. The first group containing 21 individuals varied in GS from 41 pg to 46.05 pg, with a mean of 43.14 pg (σ =1.33 pg). The second group consists of 9 shrubs with a measured GS from 21.39 pg to 22.48 pg and a mean of 22.09 pg (σ =0.35 pg).

Detailed values for each sample, indicating also the quality of the measurement by the coefficient of variation (CV %), are represented in the additional file 1.

### Hybridization between *J. sabina* and *J. thurifera* in sympatry

The chloroplast (cp) region trnS-trnG, amplified for all samples, generated sequences with 835 bp. We found 6 fixed single nucleotide polymorphisms (SNPs) and 2 indels between the cp sequences of *J. thurifera* (referred to thu cp Type) and *J. sabina* var. *sabina* (referred to sab cp Type) (Table 1). Surprisingly, all the putative hybrids (*J. x cerropastorensis*) found in the E Iberian Range populations had the same cp sequence as *J. thurifera* (thu cp Type).

**Table 1:**
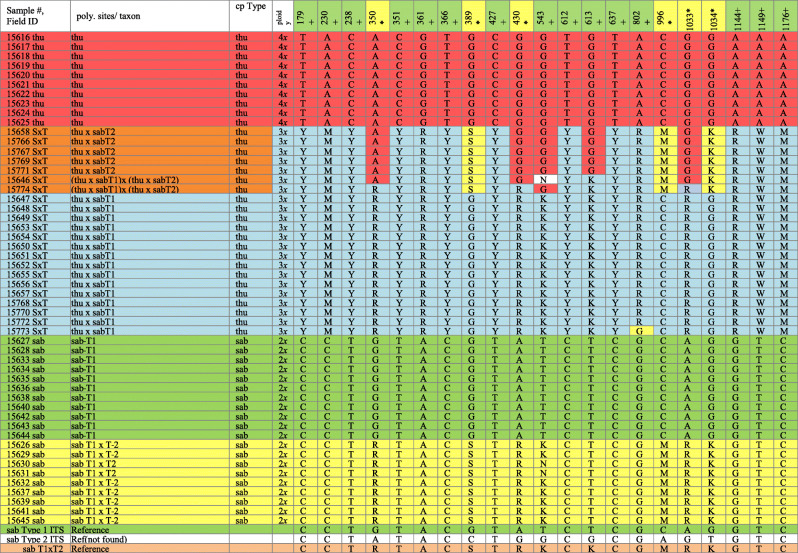
Informative SNPs (21) from Eastern Iberian Range populations. Sites in row one in yellow distinguish Type 1 (T1) and Type 2 (T2) ITS in *J. sabina* (sab) and sites in green distinguish *J. thurifera* (thu) and *J. sabina*.

Sequencing the nuclear DNA (nrDNA) region, Internal Transcribed Spacer (ITS) generated 1270–1273 bp and revealed 23 informative SNPs and one indel at site 801(deletion in *J. sabina* var. *sabina* or an addition in *J. thurifera*). It is interesting that the same deletion at 801 occurs in the nrDNA of *J. sabina* var. *balkanensis*. Two of the SNPs were very near site 801 and could not be consistently scored, and were not utilized, resulting in 21 informative SNPs (Table 1). ITS data resolved the samples from E Iberian Range populations into five groups (Table 1): 1. Ten individuals of *J. thurifera* (thu) (red, Table 1); 2. Eleven *J. sabina* var. *sabina*, Type1 haplotype (sabT1), which are very uniform (green, Table 1); 3. Five hybrids *J. thurifera* x *J. sabina* var. *sabina* (Type 2) (referred as thu x sabT2) (orange, Table 1); 4. Fifteen hybrids, *J. thurifera* x *J. sabina* var. *sabina* (Type 1) (referred as thu x sabT1) (blue, Table 1); 5. Nine homoploid *J. sabina* hybrids between the Type 1 and the Type 2 (referred as sab T1xT2) (yellow, Table 1). In addition, two plants 15646 and 15774 seem to be probably hybrids between individuals of group 3 and 4. It should be noted that pure *J. sabina* var. *sabina*, Type 2 haplotype (sabT2) was not found among these samples (Table 1, bottom). However, because the T2 haplotype is present in the sab T1 x T2 and thu x sabT2 samples, this implies that *J. sabina* var. *sabina* (Type 2) is present in the population or nearby, even though we did not collect it in this study.

**Fig. 2:**
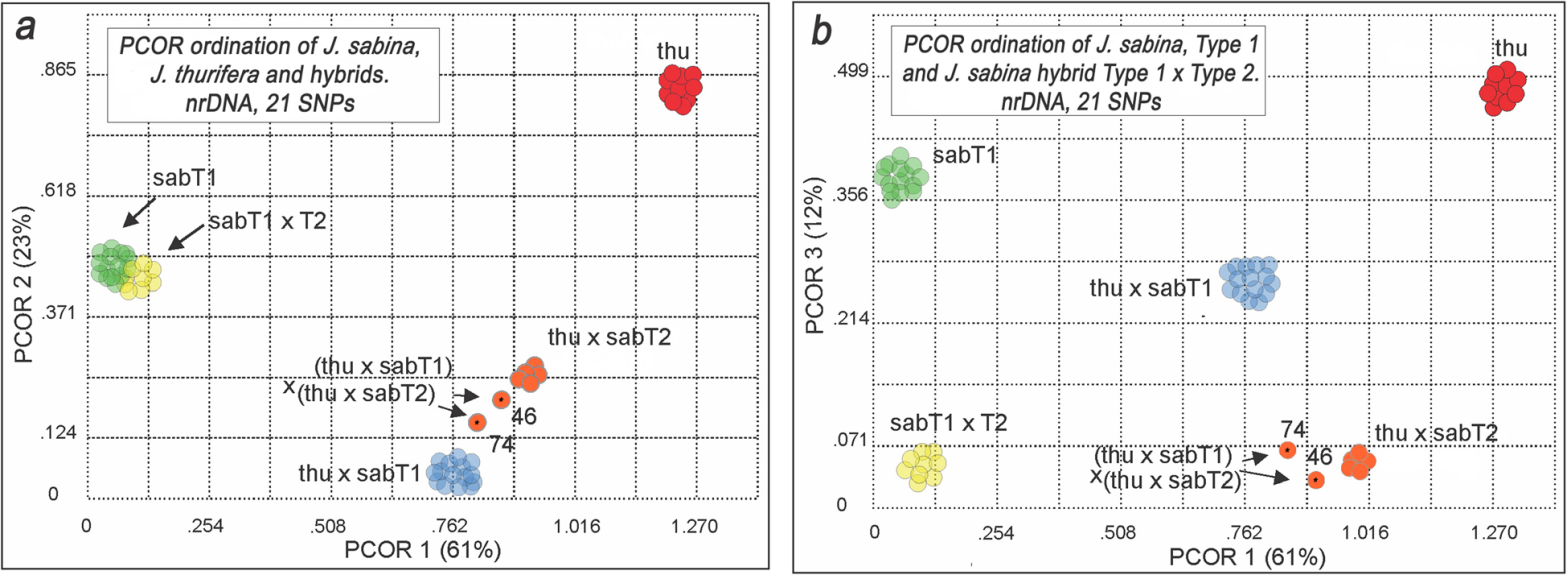
PCOR of *Juniperus* studied taxa from Eastern Iberian Range using 21 SNPs of nrDNA data. This PCOR includes 10 *J. thurifera*, 20 *J. sabina* var. *sabina*, and 22 putative hybrids. Colors correspond to Table 1

Principle Coordinates Ordination (PCOR) of the ITS data was conducted by coding the presence / absence of each allele to produce a similarity matrix among the samples. Factoring the matrix resulted in four eigenroots before asymptoting [20]. This supports the presence of five groups (# eigenroots + 1), as seen in Table 1 with the two hybrids 15646 (46) and 15774 (74) are loosely grouped with other hybrids (Fig. 2 a, b). Coordinate axis 1 (PCOR 1) separated *J. sabina* var*. sabina* from *J. thurifera* (Fig. 2a, b). The hybrids, thu x sabT1, ordinate in an intermediate position on PCOR 1 between the parents, while the PCOR 2 ordinates the hybrids as a third entity (I.e., *J. thurifera*, *J. sabina* and hybrids). Thus, the 2D ordination produces the U or V shape commonly found in the analyses of synthetic and natural hybridization [21, 22]. The five hybrids putative (thu x sabT2) are near the position of the hybrids thu x sabT1 between *J. sabina* and *J. thurifera* on the PCOR1. Notice that the samples 15646 (46) and 15774 (74) are located between the hybrids thu x sabT1 and thu x sabT2. Those two individuals are putative hybrids between those two groups as shown in the Table 1.

*Juniperus sabina* var*. sabina* (T1) and *J. sabina* var*. sabina* hybrids (T1xT2) are resolved on coordinate axis 3 (12% of the variation, Fig. 2b).

### Dynamics between *J. sabina* var. *sabina* and *J. sabina* var. *balkanensis* in a population in the Sierra de Baza: gene flow from distant, allopatric *J. thurifera*

Analyses of trnS-G (cp DNA) confirmed that all the tetraploids found in this population have cpDNA of the *J. thurifera* (=cpDNA of *J. sabina* var. *balkanensis*) Type (Table 2).

**Table 2:**
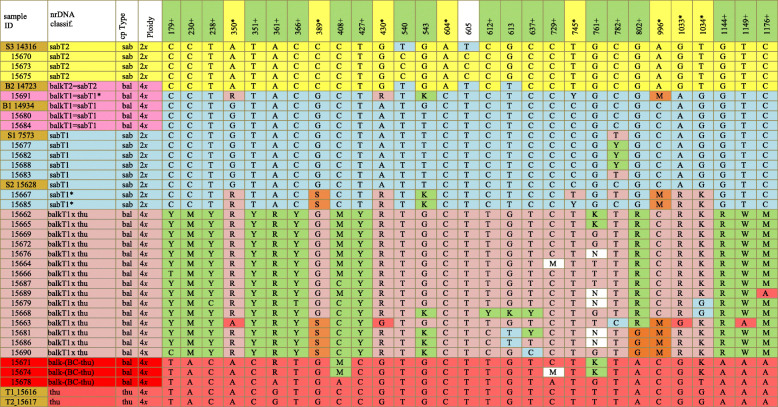
Informative SNPs (29) in the Sierra de Baza population. In row one, sites in yellow are informative about Type 1 or Type 2 ITS and sites in green and with a + are informative about hybridization between *J. sabina* and *J. thurifera*; sites with no color shading are not clearly informative or not scored (N). Several reference samples (in gold shading sample ID) are included: S3 14316 (sabT2); B2 14723 (balkT2); B1 14934 (balkT1); S1 7573 (sabT1); S2 15628 (sabT1); T1 15616 (thu); T2 15617 (thu).

Abbreviations

bal, balk: *J. sabina* var. *balkanensis,* sab: *J. sabina* var. *sabina,* thu: *J. thurifera, BC* Back cross, SxT: hybrid, *J. sabina* x *J. thurifera*

**29 Poly. Sites locations:**

1 (179), 2(230), 3(238), 4(350), 5(351), 6(361), 7(366), 8(389), 9(408), 10(427), 11(430), 12(540), 13(543), 14(604), 15(605), 16(612), 17(613), 18(637), 19(729), 20(745), 21(761), 22(782), 23(802), 24(996), 25(1033), 26(1034), 27(1144), 28(1149), 29(1176). 801 poor skipped.

ITS sequences of three *J. sabina* var. *sabina* and two *J. sabina* var. *balkanensis* as well as two *J. thurifera* samples were included in the analyses (shaded in gold, Table 2) and 29 informative sites were discovered. Both Types 1 and 2 ITS sequences were found in the population (Table 2), with the majority of plants being Type 1, similar to that reported in other regions [15–17]. Of the non-hybrid plants, three *J. sabina* var. *sabina* were Type 2 (T2), and six were Type 1 (T1). Of *J. sabina* var. *balkanensis* plants, none were Type 2, but three were Type 1 (Table 2).

Based on ploidy, cpDNA (trnS-G) and ITS sequences, the 30 samples could be divided into five groups: 1. *J. sabina* var. *sabina* (T2) (sabT2) (yellow, Table 2); 2. *J. sabina* var. *balkanensis* (T1) (balkT1) (pink, Table 2); 3. *J. sabina* var. *sabina* (T1) (sabT1) (blue, Table 2); 4. *J. sabina* var. *balkanensis* (T1) x *J*. *thurifera* putative hybrids (referred as balkT1 x thu) (salmon-beige, Table 2); and 5. Putative *J. sabina* var. *balkanensis* (T1) x *J*. *thurifera* hybrids*,* backcrossed to *J. thurifera* (referred as balk-(BC-thu)) (red, Table 2).

It is obvious that the hybrids are rather variable, and it seems likely that some of these are F_2_ progeny. Many of *J. sabina* var. *sabina* plants have one or more sites that reflects past hybridizations between the ITS Types (1 and 2).

**Fig. 3:**
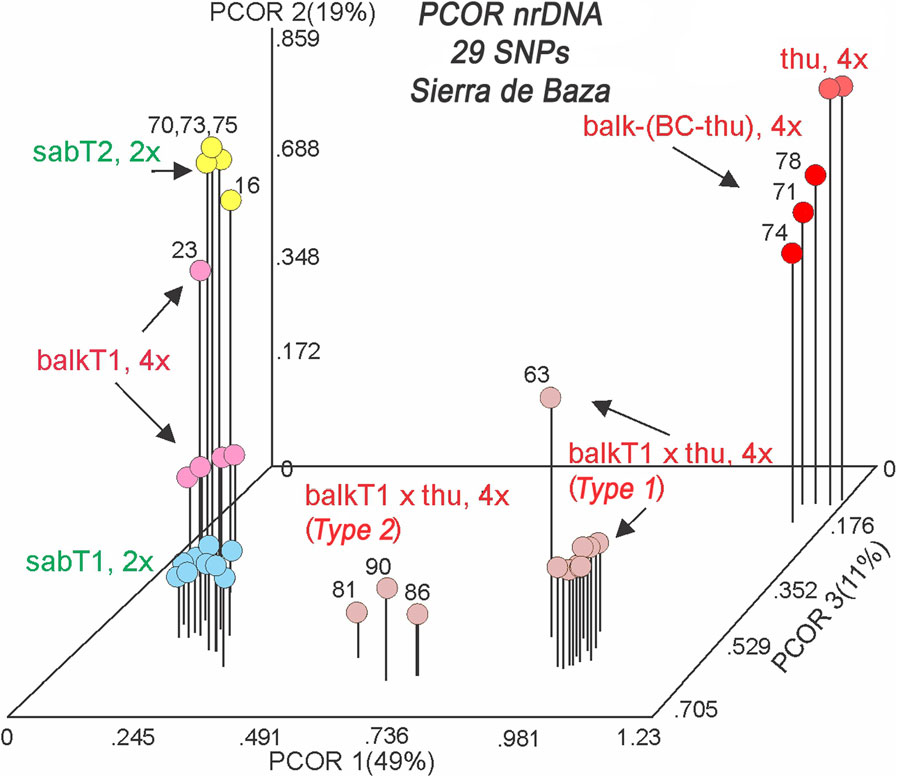
PCOR of *Juniperus* studied taxa from Sierra de Baza using 29 SNPs of nrDNA data. The PCOR ordination includes 30 *J. sabina* var. *sabina* and *J. sabina* var. *balkanensis* plus two reference *J. thurifera* samples. The group colors correspond to Table 2

Factoring the Sierra de Baza plants ITS data matrix (based on 29 SNPs) resulted in five eigenroots that accounted for 49.20, 19.22, 11.35, 4.78 and 4.10% (88.63% total) of the variance among samples. PCOR ordination using the first three coordinates reveals (Fig. 3) the first axis primarily resolved *J. sabina* (i.e., both *J. sabina* var. *balkanensis* and *J. sabina* var. *sabina*) from the reference *J*. *thurifera* (thu) samples and partially resolved *J. sabina* var. *balkanensis* x *J. thurifera* hybrids (balkT1 x thu, 4*x*) into two groups (Fig. 3). The difference between the (15681 (81), 15686 (86), 15690 (90)) group and the larger group of hybrids is readily seen in Table 2, as 81, 86, and 90 share several SNPs (note sites 389, 543, 802, 1033) that differ from the other hybrids. Sample 63 (15663) is likely F_2_ generation plant (note the variation among SNPs, Table 2). All of *J. sabina* var. *sabina*, Types 1 (sabT1, 2*x*) and 2 (sabT2, 2*x*), along with *J. sabina* var. *balkanensis* Type 1 (balkT1, 4*x*), are ordinated on the left (Fig. 3), but coordinate axis 2 resolves these groups. Axis 3 serves to separate individuals and partially resolve the (sabT1, 2*x*) group (blue, Fig. 3). It should be noted that *J. sabina* var. *balkanensis* backcrosses to *J. thurifera* (balk-(BC-thu)) group (Fig. 3) are ordinated near to the reference *J. thurifera* (thu) samples, indicative of their backcross nature.

The examination of hybridization by the use of all 29 SNPs is hindered by the inclusion of the 8 SNPs that distinguish *J. sabina* Types 1 and 2 ITS sequences. The 8 SNPs plus 4 with low information content (12 SNPs total) were removed (compare Tables 2, 3) and the data set was re-run using 17 informative SNPs. Factoring the similarity matrix yielded four eigenroots before the roots asymptoted. These four eigenroots accounted for 65, 15.19, 7 and 4.05% (90.54%) of the variance among the samples. It should be mentioned that the variance accounted for is now much larger on axis 1, 49% vs. 65%; with a reduction on axis 2: 19% vs. 15%; and axis 3: 11% vs. 7%.

**Table 3:**
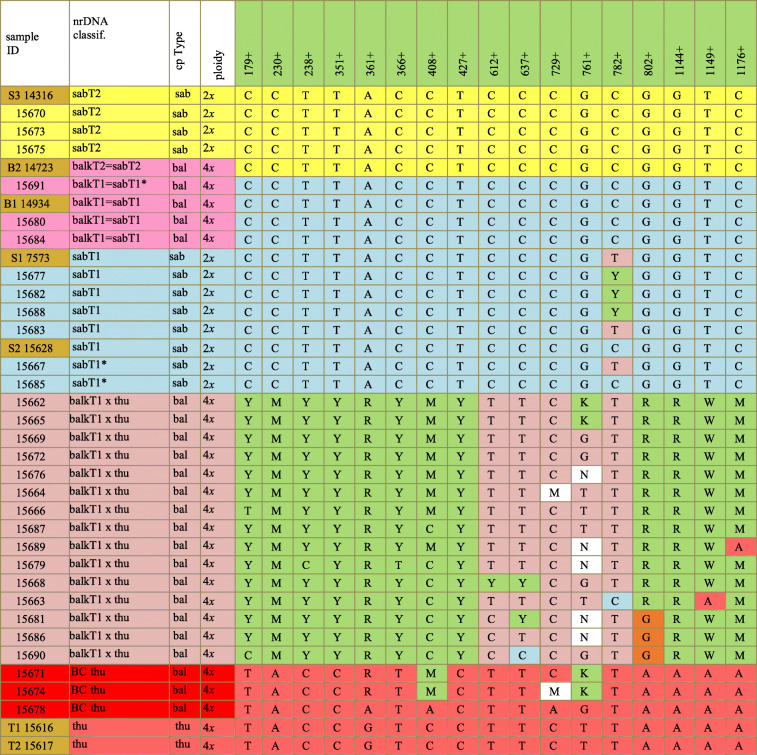
Reduced character set of 17 informative SNPs in the Sierra de Baza population. SNPs that distinguish Type 1 and 2 ITS, as well as ambiguous SNPs have been removed. Notice the uniformity of *J. sabina* var. *sabina*, Type 2 (sabT2), *J. sabina* var. *balkanensis*, Type 1 and 2 (balkT1, balkT2) groups and *J. sabina* var. *sabina* Type 1 (sabT1), except for site 782, which is very near the indel at 801.

**Fig. 4:**
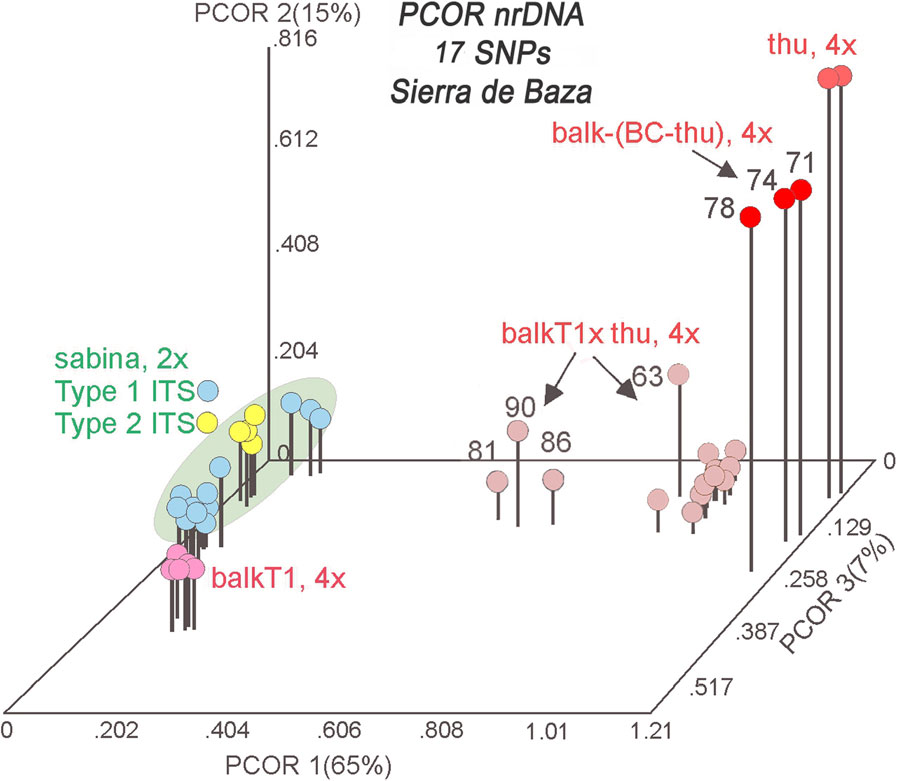
PCOR of *Juniperus* studied taxa from Sierra de Baza using 17 SNPs of nrDNA data. The PCOR ordination includes 30 *J. sabina* var. *sabina* and *J. sabina* var. *balkanensis* plus two reference *J. thurifera* samples. Note the clear separation of *J. sabina* var. *sabina* and *J. sabina* var*. balkanensis* from *J. thurifera* and the hybrids *J. sabina* var. *balkanensis* x *J. thurifera*

Ordination revealed that axis 1 clearly separates the two varieties of *J. sabina* from *J. thurifera* reference samples, and from *J. sabina* var. *balkanensis* x *J. thurifera* hybrids (balkT1 x thu, 4*x*) (Fig. 4). The hybrids now form a tighter group, but with some variation that is likely due to the presence of F_2_ generation individuals. Notice that the ITS sequence of *J. sabina* var. *balkanensis* Type 1 (balkT1, 4*x*) places it in association with *J. sabina* var. *sabina* Types 1 and 2 (Fig. 4). In fact, it is essentially unresolved by these 17 SNPs from *J. sabina* var. *sabina*. The full ITS (1270 bp) sequence provides only minor resolution between *J. sabina* var. *sabina* and *J. sabina* var. *balkanensis* (ddRAD sequencing has provided resolution, but small, between the varieties, *manuscript in preparation,* RPA).

Individual 63 (15663, Table 2) is an oddity in its loose grouping and in it several anomalous SNPs (Table 2). It may be an usual F_2_ or a backcross plant.

## Discussion

### Ploidy levels

Despite the rarity of polyploidy in conifers, a highly interesting spectrum of ploidy levels has been recently shown in wild populations of *Juniperus* genus (2*n* = 2*x*, 2*n* = 4*x* and 2*n* = 6*x*) [7, 16]. This makes polyploidy an important evolutionary mechanism which was implicated at least 10 times during *Juniperus* evolution [7]. The ploidy levels of the taxa in this study except for the potential hybrid trees were previously published [7, 16, 23, 24]. However, intra-specific variation in the ploidy level has been reported in this genus notably in *J. sabina*, *J. chinensis* L., and *J. seravschanica* Kom., [7, 16, 23–25] which makes essential the ploidy level determination of the studied populations.

In *Juniperus*, it has been shown that ploidy level can be inferred from genome size [7]. Based on this inference, in the E Iberian Range, all studied individuals of *J. thurifera* and *J. sabina* var. *sabina* were found to be tetraploid (2*n* = 4*x*) and diploid (2*n* = 2*x*), respectively. Genome sizes of the 22 putative hybrids (*Juniperus x cerropastorensis*) were intermediate between the ranges of GS defined for diploid and tetraploid levels. Therefore, those putative hybrids appear to be triploids (2*n* = 3*x*). Despite the fact that triploids are usually unstable and sterile, they are an important pathway “triploid-bridge” for reaching a stable ploidy level [26]. In a general context, many pathways were suggested to achieve a triploid stage such as polyspermy and unreduced gamete [27, 28]. In the present study, a fertilization between a diploid gamete (*n* = 2*x*) produced normally by the tetraploid *J. thurifera* and a haploid gamete from the diploid *J. sabina* var. *sabina* will produce a triploid progeny.

Recently, the first three *Juniperus* triploid hybrids between *J. thurifera* and *J. sabina* var. *sabina* have been identified in the wild in the French Alps [18]. It suggests that when *J. thurifera* and *J. sabina* var. *sabina* co-occur in the same geographical zone they may hybridize generating triploid hybrids.

In the Sierra de Baza site, containing *J. sabina* var. *sabina* and *J. sabina* var. *balkanensis*, approximately two-thirds of the shrubs showed a mean GS indicative of a tetraploid and just one-third had a GS at the diploid level. In this site, no triploids were found. Despite that *J. sabina* var. *sabina* and *J. sabina* var. *balkanensis* can scarcely be identified in the field, two criteria do separate those two taxa. The first criterion is that *J. sabina* var. *balkanensis* has the chloroplast sequences of *J. thurifera* [15, 17, 29]. The second criterion is, at present, all populations studied of *J. sabina* var. *sabina* and *J. sabina* var. *balkanensis* have been shown to be diploid and tetraploid, respectively [16]. This suggests, based on ploidy data, that 2/3 of the individuals in the Sierra de Baza population are possibly *J. sabina* var*. balkanensis* and 1/3 are probably *J. sabina* var. *sabina*.

### Homoploid and heteroploid hybridization in Eastern Iberian Range populations

The importance of interspecific hybridization as a driver for plant evolution has been debated for decades [30–33]. Lately, the significance of this phenomenon in plant speciation and evolution has been well defended [34, 35]. The usage of nrDNA (especially ITS region) to detect hybrids has been widely implemented in plant studies due to its remarkable proprieties; we mention the homogenous paralogs within individuals as a result of concerted evolution [36, 37]. As well, this marker was highly explored in nearly all *Juniperus* taxa (including haplotypes within taxa) for phylogenetic purposes and showed high efficiency in junipers hybridization studies with relatively good number of informative SNPs [5, 6, 8, 10, 11, 15, 17, 21, 29]. In this study, we found two categories of hybrids, homoploid and heteroploid hybrids. Homoploid hybrids (2*n* = 2*x*) were found between the two types of diploid *J. sabina*. It should be noted that nrDNA sequences in *J. sabina* are polymorphic, designated as Type 1 and Type 2 [15, 17, 29, 38], comprising 8 SNPs. The most recent survey of Type 1 (T1) and Type 2 (T2) ITS occurrences [17] examined 66 *J. sabina* samples from throughout the known range of *J. sabina* var. *sabina* and *J. sabina* var. *balkanensis* and found to be 27.3% Type 1, 4.5% Type 2 and 68.2% intermediate (T1 x T2 hybrids and backcrosses). Thus, it was important to determine the presence of T1 and T2 in the Spain putative hybrid populations, as this could skew the analysis of hybridization between *J. thurifera* and *J. sabina* var*. sabina*. In addition, two sets of heteroploid hybrids (2*n* = 3*x*) have been identified, both of them involving *J. thurifera* (2*n* = 4*x*) as one parent and the second parent being *J. sabina* Type 1 or Type 2 (2*n* = 2*x*).

Interestingly, among our samples, we did not find *J. sabina* var*. sabina* Type 2, but by finding the categories of hybrids cited above, it is very probable that Type 2 plants are present in a nearby population. It should be noted that, at present, nrDNA Type 1 and 2, are not associated with any morphological trait nor terpenoid(s) in the leaf essential oils [39], and thus, could not be selected during our sampling. The discovery that the two types of *J. sabina* hybridize and both could hybridize with *J. thurifera* would increase the genetic diversity of the offspring individuals and they may exhibit a reproductive isolation from the parental species leading to the speciation [40, 41]. In the E Iberian Range populations, we did not found backcrosses from the triploid hybrid with any of the parental species, which suggest the presence of a breeding barrier between the triploids and the parental species. Nevertheless, we found two triploid hybrids (15646 and 15774) that are probably the offspring generated from a cross between two triploid hybrids (thu x sabT1) x (thu x sabT2) based on their SNPs. This means that a fertilization was probably between a haploid gamete (*n* = 1*x*) with a diploid gamete (*n* = 2*x*) produced by the triploid parental hybrids. Hybridization between two triploids giving rise to a triploid hybrid is quite rare due to the high sterility and meiotic problems of triploid hybrids, as described in many studies [26]. However, it has been shown that triploids are not completely sterile and could produce some viable gametes of three ploidy levels (*n* = 1*x*; *n* = 2*x* and *n* = 3*x*) [26, 42, 43]. In *Juniperus*, the production of reduced, partially reduced and unreduced male gametes has been suggested for the triploid hybrids found in the Alps based on the significant variation of pollen sizes [18]. Interestingly, those hybrids, found very recently in the French Alps, were also between *J. thurifera* and *J. sabina* var. *sabina* present in a sympatric occurrence [18]. The hybridization events found between *J. thurifera* and *J. sabina* in the French Alps (France) and in the E Iberian Range (Spain), suggest that the reproductive barriers between those two species are ineffective despite the difference of ploidy levels.

All triploid hybrids found in this study shared the same chloroplast marker trnS-G which is the *J. thurifera* cp Type. Preliminary analysis of four cp DNA markers (petN-psbM, trnS-G, trnL-F and trnD-T) revealed that all four distinguished *J. sabina* from *J. thurifera* [5, 15, 17], but cp marker trnS-G yielded the largest number of informative SNPs. Chloroplasts appear to be inherited via pollen in *Juniperus,* because the chloroplasts of Cupressaceae species examined to date have been shown mainly to be paternally inherited [44]. Thus, all 22 hybrids were derived from unidirectional crosses involving male (pollen) *J. thurifera* trees. The unidirectional crossing seems to imply *J. thurifera* has evolved a reproductive barrier against *J. sabina* pollen, but not vice-versa. Unidirectional interspecific hybridization has been reported in several genera in angiosperms [45–47] and in gymnosperms, especially in *Pinus* L. [48] and in *Juniperus* [49]. Lepais et al. [50], suggested that unidirectional gene flow in sympatry could be affected by the relative abundance of species where introgression will be from the more frequent species to the less frequent one. However, it seems that this hypothesis is not valid in the two cases of hybrids found in the French Alps and Spain, due to the approximately similar abundance of both *J. sabina* var. *sabina* and *J. thurifera* in those locations. However, the unidirectional hybridization could be due to the difference in timing of reproduction between those two species. *Juniperus thurifera* sheds pollen in the winter but *J. sabina* var. *sabina* sheds pollen in the late winter till spring [6]. Usually, flowering and shedding pollen co-occur to insure the good reproduction in dioecious species which is the case of both *J. sabina* var. *sabina* and *J. thurifera*. See section below for extensive discussion about overlapping pollen shedding times.

Interestingly, in contrast to angiosperms where crosses seem to be more successful when the maternal parent has a higher ploidy level [26], in the studied *Juniperus*, the paternal parent was shown to be tetraploid and the maternal parent as diploid in all triploid hybrids. The main factor implicated in the inter-ploidy hybridization in angiosperms is the endosperm maternal/ paternal ratio that was destructive when the paternal parent had a higher ploidy level than the maternal parent leading to an aborted seed [26]. The fact that there is no endosperm in *Juniperus* this could be one of the reasons of the interspecific hybridization success regardless of the maternal and paternal parents’ ploidy levels. However, this hypothesis must be taken with high caution because no research has been done to date to study the presence of genetic barriers that prohibit the hybridization between a female *J. thurifera* (2*n* = 4*x*) and a male *J. sabina* (2*n* = 2*x*). Clearly, further studies are needed in this field.

### Allopatric introgression in the Sierra de Baza population

Allopatric hybridization and introgression in *Juniperus* have been frequently reported [9–12, 15, 51–54]. So, it is not surprising to find the DNA data clearly supports introgression by pollen from allopatric *J. thurifera* into *J. sabina* var. *balkanensis*, Type 1 (Figs. 3, 4) in the Sierra de Baza population. In the present population, based on the ploidy and the results of trnS-G and ITS, those tetraploid hybrids may arise from a fertilization between normally reduced diploid gamete (*n* = 2*x*) from the tetraploid taxa *J. thurifera* and *J. sabina* var. *balkanensis*. We couldn’t distinguish between male and female parental taxa because *J. thurifera* and *J. sabina* var. *balkanensis* have the same chloroplast sequences [15, 29, 38]. We expected to find triploid hybrids between *J. sabina* var. *sabina* (2*x*) and *J. sabina* var. *balkanensis* (4*x*) at Sierra de Baza in an analogous fashion as we found in the E Iberian Range populations where there were triploid hybrids between *J. sabina* var*. sabina* and *J. thurifera*. In E Iberian Range populations, *J. thurifera* and *J. sabina* var. *sabina* grew intermingled on hillsides, and triploids were found scatter among them. At the Sierra de Baza population, *J. sabina* var. *sabina* and *J. sabina* var. *balkanensis* grew intermingled. Yet no triploids were found. There may have developed some isolating mechanism(s) to prevent hybridization between these varieties which could be a strategy to speciation in the case of *J. sabina*. In fact, reproductive isolation was shown to play a central role leading to speciation with pre or post-zygotic barriers [26, 55–57].

In contrast to the E Iberian Range populations, where it appears that most hybrid individuals were holding highly similar ITS sequences suggesting being first generation, at Sierra de Baza, most tetraploid hybrids showed genetic diversity suggesting to be more as F2 or higher generation level backcrossed to *J. thurifera* as obviously observed in 15671–15674 and 15678 hybrids. This could be due to the high gametes abortion in triploid hybrids due to unbalanced meiotic chromosome segregation and numerous meiotic abnormalities we cite the precocious cytokinesis found in allotriploid poplar [43]. Meiosis might be more stable or reaching faster a stable condition in allo-tetraploid than in allo-triploid. This would be one of the reasons to find more diversity in allo-tetraploid hybrids produced in “one step” between two tetraploid parental species than the allo-triploid hybrids that could have more serious problems in meiosis. Interestingly, in this population we found backcrosses of the hybrids with *J. thurifera* and none with *J. sabina* suggesting an unidirectional gene flow as observed in E Iberian Range populations. Unfortunately, we don’t have data for pollen and flowering periods of *J. sabina* var. *balkanensis* and the hybrids found, to check possible pre-zygotic reproductive barriers. However, further work will be dedicated to discover the reproductive barriers between this group of taxa.

### Overlapping pollen shedding/ receptive female cones seasons

Literature reports of pollen shedding times for *J*. *thurifera* vary from: winter [6]; flower(s) by January [58]; flower(s) at the end of winter period [59]; late winter-early spring [60], between January and May [61, 62].

Pollen shedding time for *J. sabina* has been reported as late winter-spring [6], April ([61, 62], mid-March-April (French Alps, pers. comm. Thierry Robert), April–May (Bulgaria, pers. comm., Alex Tashev). A summary for *J. thurifera* depicts (Table 4) the prime or most likely pollen shedding times are in red (January, February). Less likely pollen shedding times are in orange and rarely occurring times in yellow (Table 4) and likewise for *J. sabina*. Note that the overlapping times of major pollen release (*J. thurifera*) and major receptive cones of *J. sabina* are February–March (Table 4). Thus, clearly these taxa have an overlapping season for gene exchange (as demonstrated by the production of hybrids in the E Iberian Range populations).

**Table 4:**
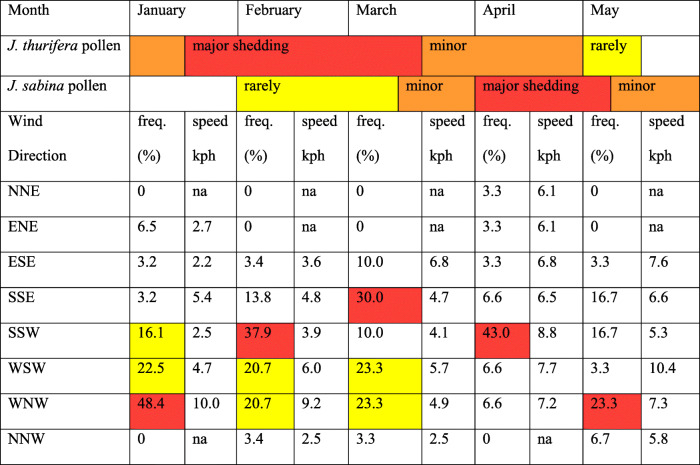
Data for wind direction, frequency (freq.) (%), and velocity (kph = km/h) along with major (red) and minor (yellow) pollen shedding times for *J. sabina* and *J. thurifera*. Wind data from city of Baza, Spain [63]. Times of receptive cones are highly correlated with pollen shedding (time) in *Juniperus* [60]. na = not applicable. North-northeast (NNE), east-northeast (ENE), east-southeast (ESE), south-southeast (SSE), south-southwest (SSW), west-southwest (WSW), west-northwest (WNW) and north-northwest (NNW).

**Fig. 5:**
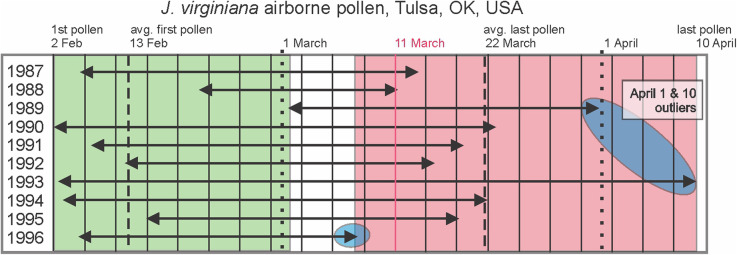
Start and ending dates for airborne pollen of local *J. virginiana* at Tulsa, OK for ten years. Green box includes all the start dates of *J. virginiana* pollen captured and Red rectangle encompasses the ending dates. Adapted from data in [64]

Even if pollen shedding times scarcely overlap, given many years of seasons and that pollen shedding times vary from year to year, occasionally, pollen shedding will likely overlap. An exceptional study on variation in pollen shedding times for *J. virginiana* airborne pollen over a 10-year period [64] found (Fig. 5) that the start of pollen shedding varied from 2 February (1990) to 13 February (1992). The termination of pollen shedding was very variable from March 11 to April 10 (Fig. 5). Notice that 1988 was an unusual year in having a very short pollen shedding time (February 21–March 11, 19 days of airborne *J. virginiana* pollen). And, 1993 had an exceptionally long season from February 3–April 10, 67 days of airborne pollen. Pollen shedding (release) in late winter and spring is dependent on two temperature factors [64]: chilling requirement to end dormancy [65] and accumulation of heat units above a threshold temperature [65, 66]. Pollen is released from junipers on warm, dry days and often one will see a sudden yellow, pollen cloud rising above a juniper tree as the morning sun warms and dries the air and pollen cones.

Pertinent to this discussion is the fact that a significant amount of *Juniperus* pollen can travel very long distances by wind. For example, major concentrations of *J. ashei* J. Buchholz pollen (shed in December–January) are blown to Tulsa, Oklahoma from the nearest major pollen source, 320 km south, in southern Oklahoma and Texas.

Levetin [64] notice (Table 5) that in 1993–94 and 1995–96, maximum concentrations of *J. ashei* pollen were greater than for the local *J. virginiana*. This shows that even after traveling more than 320 km, the concentration of distant pollen can be on the same level as local pollen. Recently, it has been proven by DNA analysis of individual pollen grains, that *J. ashei* from Texas traveled to and was collected in Ontario, Canada, approximately 2400 km [67].

**Table 5:** Comparison peak concentrations of airborne *J. ashei* pollen (December–January) to *J. virginiana* pollen (February–March) captured at Tulsa, OK. * = concentration of invasive pollen higher than local pollen

	Peak concentration, pollen grains/m^3^		
year (season)	*J. ashei,* from southern Oklahoma, Texas	*J. virginiana* from local Tulsa area	foreign (*J. ashei*) vs. vs. local (*J. virginiana*) pollen
1987–88	175	655	0.27:1
1988–89	546	1057	0.51:1
1989–90	257	1115	0.23:1
1990–91	333	4442	0.07:1
1991–92	158	1156	0.14:1
1992–93	802	1248	0.64:1
1993–94	2027	1311	1.55:1*
1994–95	947	1485	0.64:1
1995–96	2411	2027	1.19:1*

Several other studies in conifers have reported long distance transport (LDT) of pollen from a few km to several hundred km [68–74]. Importantly, several studies have reported that LDT pollen has maintained its viability [75–77]. Pollen from *Juniperus communis* L., in the western Alps, was stored at ambient conditions and found to be 40–90% viable in fresh pollen, 20–40% viable after 2 weeks and 0–10% viable after 2 months storage [78].

At present, very few field studies in conifers have shown that LDT viable pollen is effective (that is, able to fertilize receptive strobili). However, in a small isolated population of *Pinus sylvestris* L., in Spain, effective pollen was discovered 100 km from the source at a rate of 4.4% [79, 80]. Molecular markers (4 chloroplast and nuclear microsatellites (SSR) were used to perform paternity tests on 813 seeds. Although 778 seeds had fathers of local origin, 4.3% (35) seed fathers were from immigrant LDT pollen [80].

Finally, it should be mentioned that in a preliminary study on LDT pollen viability, Levetin (per. Comm.) has found viable *Juniperus* (*J. ashei*) LDT airborne pollen in Tulsa after having traveled at least 320 km from southern Oklahoma, Texas.

### Potential source of *J. thurifera* nearby the Sierra de Baza *J. sabina* var. *balkanensis* population

**Fig. 6:**
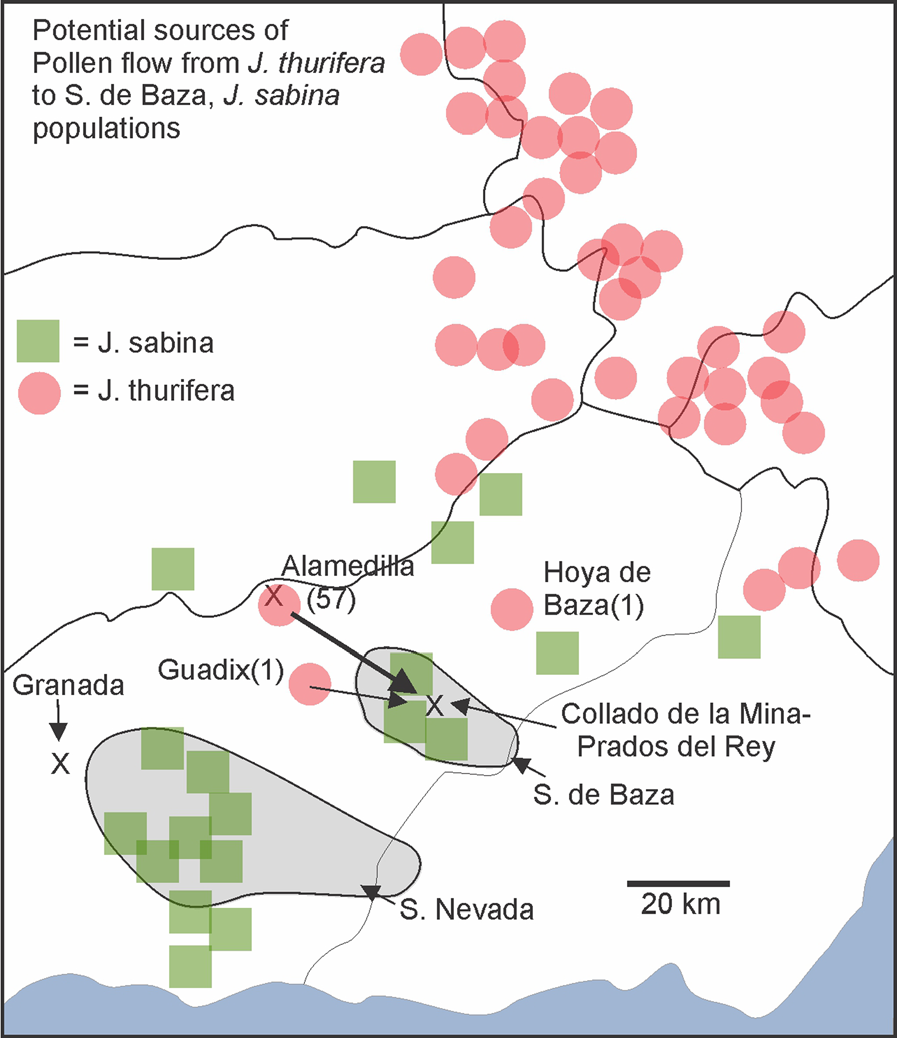
Distribution of *J. thurifera* and *J. sabina* in the region around Sierra de Baza (S. de Baza). Data from Anthos Spanish Plants Information System (http://www.anthos.es/index.php?lang=en) and FAME database [81]. Numbers in parenthesis are the number of records in FAME database for that location (ex. Alamedilla (57) denotes 57 records at that location)

Examination of distributions of *J. thurifera* and *J. sabina* in the area (Fig. 6) reveals several *J. thurifera* populations Northeast of Sierra de Baza and a significant population of *J. thurifera* in the Alamedilla area, west-northwest (WNW) of Sierra de Baza. Although FAME database contained only 57 records from the Alamedilla area, there are likely many more trees around Alamedilla than 57. In the cases of Guadix (1) and Hoya de Baza (1), these sites each have only a single, isolated tree (personal observation, Carlos Salazar-Mendias (CSM)). The nearest large population of *J. thurifera* is Alamedilla that is approximately 26 km from *J. sabina* var. *balkanensis*, Sierra de Baza (Fig. 6).

Wind direction and velocity are essential factors for LDT of pollen grains that are dispersed by wind which is the case in *Juniperus*. Analyses of wind directions and velocities for January, February, March, April and May show (Table 4) the major directions are from: WNW, 48.4%, 10.0 kph (January); south-southwest (SSW), 37.9%, 3.9 kph (February); south-southeast (SSE), 30.0%, 4.7 kph (March), SSW, 43%, 8.8 kph (April) and WNW, 23.3%, 7.3 kph (May). Overall the wind velocities were not very large, ranging from 2.2 to 10.0 kph.

**Fig. 7:**
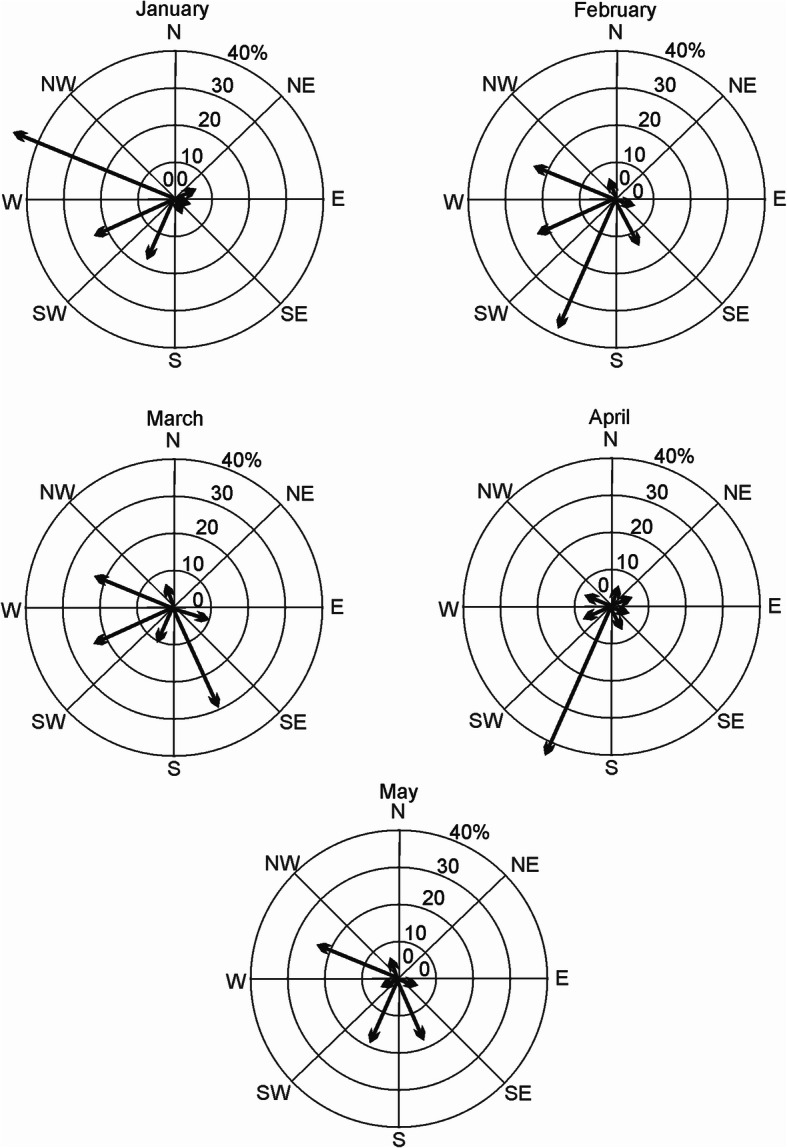
Frequencies of wind directions, Baza, Spain for January, February, March, and April. The most dominant directions were from WNW, 48.4% (January) and SSW, 43% (April). Source: (Wind data from city of Baza, Spain [63])

Graphical wind directional analyses reveal the prevailing winds for January and February are similar being from the west, northwest and southwest (Fig. 7). A second pattern emerges in March, but considerable winds blow from WNW (23.3%) and west-southwest (WSW) (23.3%), but with 30% of the winds from SSE (Fig. 7). In April, the prevailing wind pattern is clearly different as wind blows (43%) from the SSW.

The major times for pollen release from *J. thurifera* are January–February-March (note Table 4) and this coincides with the prevailing winds from Alamedilla to Sierra de Baza. Given the overlap pollen shedding (and receptive female cones), it would seem that all the factors align to support the DNA data of allopatric introgression via *J. thurifera* pollen upon receptive female strobili of *J. sabina* var. *balkanensis*, Type 1 (Fig. 4) to produce backcrossed progeny towards *J. thurifera*.

### Potential factors that interfere in the hybridization and polyploidy of the Spanish populations

Polyploidy and hybridization have been shown to be influenced by environmental and geographical factors. As an example, plant species migrate to new niches with more favorable environmental factors, thus providing a new sympatric occurrence with a sister species, which could lead to new opportunities for interspecific hybridization [82, 83]. In addition, extreme temperatures have been proven to induce unreduced gametes formation [84, 85],which is a major mechanism leading to polyploidy [1, 26]. In the present case, the polyploid taxa (*J. thurifera* and *J. sabina* var. *balkanensis*) are tetraploid in all populations studied to date [29, 86]. In the Spanish populations studied, interspecific hybridization was shown to be the driver for polyploid formation (triploid hybrids in the E Iberian Range and tetraploid hybrids in Sierra de Baza). Sierra de Baza area is subjected to LDT of *J. thurifera* pollen blown into this population appears to have led to the formation of tetraploid hybrids with *J. sabina* var. *balkanensis*. The E Iberian Range, where the populations of hybrids between *J. thurifera* and *J. sabina* occur in this study, is characterized in its central region by extreme variation in its continental climate, with wide thermal gradients between day and night (17 to 20 °C on average) and frequent thermal inversion phenomena [87]. The lowest temperatures ever recorded in inhabited areas in Spain have occurred in this geographical environment, with episodes of very cold weather that recur over periods of 6–8 years on average, in which the minimum temperatures can drop to − 20 °C, with a record low of − 30 °C in December 1963 [88]. These facts may have influenced the facilitation of hybridization processes. Normally, the optimum altitude of *J. sabina,* in the E Iberian Range is above 1600 m. However, the phenomena of thermal inversion (accumulation of cold air at the bottom of valleys in calm periods) could have favored the migration of *J. sabina* to lower altitudes (1300–1400 m) and, thus, may led to sympatric populations with *J. thurifera* in several wide-spread areas. This sympatry has facilitated interspecific hybridization between *J. sabina* and *J. thurifera*. In addition, environmental factors may interfere in the phenology of the species as reported in several studies [89, 90], and thus, led to a longer time period of reproductive overlapping. Further studies are needed on the biogeography and phenology related to environmental factors in the Spanish populations to investigate these hypotheses.

## Conclusion

This study reports the evidence of gene flow between diploid and tetraploid juniper taxa in sympatric occurrence in Spain. The populations of E Iberian Range presented triploid hybrids between the diploid *J. sabina* var. *sabina* and the tetraploid *J. thurifera*. Those hybrids were most probably of first generation. The population of Sierra de Baza showed just tetraploid hybrids, suggested to be between the two tetraploid taxa *J. sabina* var. *balkanensis* and *J. thurifera* based on ITS sequences. In this population, hybrids showed to be genetically diverse and suggested to be of F2 or higher generation level and making backcrosses with the parental species *J. thurifera*.

The studied taxa showed diversity in polyploidy pathways between the two populations; via “triploid bridge” as suggested in E Iberian Range populations and “One step polyploidy” as suggested for Sierra de Baza hybrids. However, future work on population genetics especially for Sierra de Baza population is needed using hybridization based target enrichment and NGS sequencing to study the largest number of low copy genes and to have more clear results on the hybrids backcrosses and gene flow. In addition, this further work will help in the detection of the positive selection genes and their relative functions which could be related in the adaptation and regulation of polyploid junipers.

Moreover, we showed evidence of unidirectional gene flow in both studied populations. In the E Iberian Range populations, the unidirectional gene flow showed from *J. thurifera* to *J. sabina* suggests pre-zygotic barriers related to different phenology favoring always (till now) the hybridization from *J. thurifera* to *J. sabina*. In Sierra de Baza population, the unidirectional gene flow was shown between the hybrids and one of the parental species *J. thurifera* suggesting reproductive barriers developed in the hybrids towards the parental species *J. sabina*. Yet, further work is needed to discover the mechanisms of reproductive barriers involved in the studied taxa shaping their gene flow and evolution.

## Methods

### Plant collections

Leaf samples were made from natural populations (see additional file 2). One gram (fresh weight) of the foliage was placed in 20 g of activated silica gel and transported to the lab, thence stored at -20^o^ C until 70 mg of the silica gel dried leaves was used for ploidy determination by flow cytometry. In addition, genomic DNA (10–12 mg of the silica gel dried leaves) was extracted for sequencing.

### DNA analyses

DNA was extracted from juniper leaves by use of a Qiagen mini-plant kit (Qiagen, Valencia, CA) as per manufacturer’s instructions. Amplifications were performed in 30 μl reactions using 6 ng of genomic DNA, 1.5 units Epi-Centre Fail-Safe Taq polymerase, 15 μl 2x buffer E (trnS-trnG) or K (nrDNA) (final concentration: 50 mM KCl, 50 mM Tris-HCl (pH 8.3), 200 μM each dNTP, plus Epi-Centre proprietary enhancers with 1.5–3.5 mM MgCl_2_ according to the buffer used) and 1.8 μM each primer. The primers for ITS (nrDNA) and cp trnS-trnG regions have been previously reported [91, 92]. The PCR reaction was subjected to purification by agarose gel electrophoresis. In each case, the band was excised and purified using a Qiagen QIAquick gel extraction kit (Qiagen, Valencia, CA). The gel purified DNA band with the appropriate sequencing primer was sent to McLab Inc. (San Francisco) for sequencing. 2.31 (Technelysium Pty Ltd.). Principle Coordinates (PCOR) and Minimum spanning networks software follows the formulations Veldman [20] Ordination and Adams [93].

### Genome size analyses

#### Sample preparation

Nuclear DNA amounts were assessed by flow cytometry using silica gel dried leaves of *Juniperus* samples and fresh leaves of the internal standard (IS) *Hordeum vulgare* L., cv. *‘Sultan’* (2C value = 9.81 pg) [94]. Around 30 mg of the IS and juniper leaves were simultaneously chopped in 600 ul of cold Gif nuclear isolation buffer-GNB [95]. The nuclei suspension was filtered using a nylon mesh (50 μm) and stained with 100 μg/ml propidium iodide (PI).

#### Flow cytometry analyses

DNA contents (~ 3000 stained nuclei) were determined using CytoFLEX S flow cytometer (Beckman Coulter- Life Science United States). Each sample studied was repeated twice and fluorescence signals from stained nuclei were acquired with 561 nm laser line and 610/20 nm emission filter using CytExpert 2.3 software. Analyses were performed using Kaluza Analysis 2.1 software (Beckman Coulter).To calculate the 2C DNA value, we used the formula below that study the linear relationship between fluorescence signals from stained nuclei of the IS and juniper samples.

$$ 2\mathrm{C}\ \mathrm{DNA}\ \mathrm{content}\ \left(\mathrm{pg}\right)=\left(\frac{\mathrm{Sample}\ 2\mathrm{C}\ \mathrm{peak}\kern0.17em \mathrm{mean}}{\mathrm{Standard}\ 2\mathrm{C}\ \mathrm{peak}\kern0.17em \mathrm{mean}}\right)\times \mathrm{Standard}\ 2\mathrm{C}\ \mathrm{DNA}\ \left(\mathrm{pg}\right) $$

## Supplementary information

**Additional file 1.** Genome size measurements of *Juniperus* samples.

**Additional file 2.** Collection information and field notes.

## Data Availability

DNA sequences are available from GenBank repository accessions: GenBank MT136620-MT136701 for the region: trnS-trnG intergenic spacer, complete sequence; TrnG, partial sequence; chloroplast. GenBank MT137794-MT137875 for the region: 18S ribosomal RNA gene, partial sequence; internal transcribed spacer 1, 5.8S ribosomal RNA gene, and internal transcribed spacer 2, complete sequence; and 26S ribosomal RNA gene, partial sequence.

## Abbreviations

balk, bal.: *Juniperus sabina* var. *balkanensis*
cpDNA: Chloroplast DNA
cv.: Cultivar
E Iberian Range: Eastern Iberian range
Freq: Frequency
g: Gram
GS: Genome size
IS: Internal standard
mg: Milligram
mM: Millimolar
na: Not applicable
ng: Nanogram
nrDNA: Nuclear DNA
PCOR: Principle coordinates ordination
pg: Picogram
PI: Propidium iodide
sab: *Juniperus sabina* var. *sabina*
SNP: Single Nucleotide Polymorphism
thu: *Juniperus thurifera*
T1: Type 1
T2: Type 2
ul: Microliter
var.: Variety

## References

[1] Otto SP, Whitton J (2000). Polyploid incidence and evolution. Annu Rev Genet.

[2] Goulet BE, Roda F, Hopkins R (2017). Hybridization in plants: old ideas, new techniques. Plant Physiol.

[3] Husband BC, Baldwin SJ, Suda J (2013). The incidence of polyploidy in natural plant populations: major patterns and evolutionary processes. Plant genome diversity volume 2: physical structure, behaviour and evolution of plant genomes.

[4] Ahuja MR (2005). Polyploidy in gymnosperms: revisited. Silvae Genet.

[5] Adams RP, Schwarzbach AE (2013). Phylogeny of *Juniperus* using nrDNA and four cpDNA regions. Phytologia.

[6] Adams RP (2014). Junipers of the world: the genus *Juniperus*.

[7] Farhat P, Hidalgo O, Robert T, Siljak-Yakovlev S, Leitch I, Adams RP, Bou Dagher Kharrat M (2019). Polyploidy in the conifer genus *Juniperus*: an unexpectedly high rate. Front Plant Sci.

[8] Adams RP (2017). Multiple evidences of past evolution are hidden in nrDNA of *Juniperus arizonica* and *J. coahuilensis* populations in the trans-Pecos, Texas region. Phytologia.

[9] Adams RP (2015). Allopatric hybridization and introgression between *Juniperus maritima* RP Adams and *J. scopulorum* Sarg.: evidence from nuclear and cpDNA and leaf terpenoids. Phytologia.

[10] Adams RP (2015). Allopatric hybridization and introgression between *Juniperus maritima* RP Adams and *J. scopulorum* Sarg. II. Additional evidence from nuclear and cpDNA genes in Montana, Wyoming, Idaho and Utah. Physiologia.

[11] Adams RP, Wingate D (2008). Hybridization between *Juniperus bermudiana* and *J. virginiana* in Bermuda. Phytologia.

[12] Palma-Otal M, Moore W, Adams RP, Joswiak G (1983). Morphological, chemical, and biogeographical analyses of a hybrid zone involving *Juniperus virginiana* and *J. horizontalis* in Wisconsin. Can J Bot.

[13] Terry RG, Nowak RS, Tausch RJ (2000). Genetic variation in chloroplast and nuclear ribosomal DNA in Utah juniper (*Juniperus osteosperma*, Cupressaceae): evidence for interspecific gene flow. Am J Bot.

[14] Vasek FC (1966). The distribution and taxonomy of three western junipers. Brittonia.

[15] Adams RP, Schwarzbach A, Tashev A (2016). Chloroplast capture by a new variety, *Juniperus sabina* var. *balkanensis* RP Adams and AN Tashev, from the Balkan peninsula: A putative stabilized relictual hybrid between *J. sabina* and ancestral *J. thurifera*. Phytologia.

[16] Farhat P, Siljak-Yakovlev S, Adams RP, Bou Dagher Kharrat M, Robert T (2019). genome size variation and polyploidy in the geographical range of *Juniperus sabina* L. (Cupressaceae). Bot Lett.

[17] Adams RP, Boratynski A, Marcysiak K, Roma-Marzio F, Peruzzi L, Bartolucci F, Conti F, Mataraci T, Schwarzbach A (2018). Discovery of *Juniperus sabina* var. *balkanensis* RP Adams and AN Tashev in Macedonia, Bosnia-Herzegovina, Croatia and central and southern Italy and relictual polymorphisms found in nrDNA. Phytologia.

[18] Farhat P, Takvorian N, Avramidou M, Garraud L, Adams RP, Siljak-Yakovlev S, Bou Dagher Kharrat M, Robert T. First evidence for allo-triploid hybrids between *Juniperus thurifera* and *J. sabina* in a sympatric area in the French Alps. Accepted in Ann. For. Sci. Manuscript number is AFSC-D-19-00296R3; 2020. DOI : 10.1007/s13595-020-00969-7.

[19] Aparicio JM, Uribe-Echebarria PM (2009). *Juniperus x cerropastorensis*, nuevo híbrido entre *Juniperus sabina* L. y *Juniperus thurifera* L. Toll Negre.

[20] Veldman DJ (1967). Fortran programming for the behavioral sciences.

[21] Adams RP (1982). A comparison of multivariate methods for the detection of hybridization. Taxon.

[22] Adams RP, Stoehr M (2013). Multivariate detection of hybridization using conifer terpenes II: analyses of terpene inheritance patterns in *Pseudotsuga menziesii* F1 hybrids. Phytologia.

[23] Siljak-Yakovlev S, Pustahija F, Šolić E, Bogunić F, Muratović E, Bašić N, Catrice O, Brown S (2010). Towards a genome size and chromosome number database of Balkan flora: C-values in 343 taxa with novel values for 242. Adv Sci Lett.

[24] Vallès J, Garnatje T, Robin O, Siljak-Yakovlev S (2015). Molecular cytogenetic studies in western Mediterranean *Juniperus* (Cupressaceae): a constant model of GC-rich chromosomal regions and rDNA loci with evidences for paleopolyploidy. Tree Genet Genom.

[25] Nagano K, Matoba H, Yonemura K, Matsuda Y, Murata T, Hoshi Y (2007). Karyotype analysis of three *Juniperus* species using fluorescence in situ hybridization (FISH) with two ribosomal RNA genes. Cytologia.

[26] Ramsey J, Schemske DW (1998). Pathways, mechanisms, and rates of polyploid formation in flowering plants. Annu Rev Ecol Evol Syst.

[27] Tayalé A, Parisod C (2013). Natural pathways to polyploidy in plants and consequences for genome reorganization. Cytogenet Genome Res.

[28] Husband BC (2004). The role of triploid hybrids in the evolutionary dynamics of mixed-ploidy populations. Biol J Linn Soc.

[29] Adams RP, Farhat P, Shuka L, Siljak-Yakovlev S (2018). Discovery of *Juniperus sabina* var. *balkanensis* RP Adams and AN Tashev in Albania and relictual polymorphisms found in nrDNA. Phytologia..

[30] Stebbins GL (1959). The role of hybridization in evolution. Proc Am Phil Soc.

[31] Arnold ML (2016). Anderson's and Stebbins' prophecy comes true: genetic exchange in fluctuating environments. Syst Bot.

[32] Mallet J, Besansky N, Hahn MW (2016). How reticulated are species?. BioEssays..

[33] Wagner W (1970). Biosystematics and evolutionary noise. Taxon.

[34] Rieseberg LH (1997). Hybrid origins of plant species. Annu Rev Ecol Evol Syst.

[35] Mallet J (2007). Hybrid speciation. Nature..

[36] Baldwin BG, Sanderson MJ, Porter JM, Wojciechowski MF, Campbell CS, Donoghue MJ (1995). The ITS region of nuclear ribosomal DNA: a valuable source of evidence on angiosperm phylogeny. Ann Mo Bot Gard.

[37] Giudicelli GC, Mäder G, Silva-Arias GA, Zamberlan PM, Bonatto SL, Freitas LB (2017). Secondary structure of nrDNA internal transcribed spacers as a useful tool to align highly divergent species in phylogenetic studies. Genet Mol Biol.

[38] Adams RP, Boratynski A, Mataraci T (2017). Discovery of *Juniperus sabina* var. *balkanensis* RP Adams and AN Tashev in western Turkey (Anatolia). Phytologia.

[39] Adams RP, Mataraci T, Tashev AN (2018). The composition of the leaf essential oils of *J sabina var balkanensis*: chemotypes high in trans-sabinyl acetate and methyl eugenol discovered in three natural populations. Phytologia.

[40] Grant V (1981). Plant speciation.

[41] Ramsey J, Schemske DW (2002). Neopolyploidy in flowering plants. Annu Rev Ecol Evol Syst.

[42] Schinkel CC, Kirchheimer B, Dullinger S, Geelen D, De Storme N, Hörandl E (2017). Pathways to polyploidy: indications of a female triploid bridge in the alpine species *Ranunculus kuepferi* (Ranunculaceae). Plant Syst Evol.

[43] Wang J, Huo B, Liu W, Li D, Liao L. Abnormal meiosis in an intersectional allotriploid of *Populus* L. and segregation of ploidy levels in 2*x*×3*x* progeny. PLoS One 2017;12:e0181767. https://doi.org/10.1371/journal.pone.0181767. 10.1371/journal.pone.0181767 PMC5521839 28732039

[44] Adams RP (2019). Inheritance of chloroplasts and mitochondria in conifers: a review of paternal, maternal, leakage and facultative inheritance. Phytologia.

[45] Zhou R, Gong X, Boufford D, Wu C-I, Shi S (2008). Testing a hypothesis of unidirectional hybridization in plants: observations on *Sonneratia*, *Bruguiera* and *Ligularia*. BMC Evol Biol.

[46] Field D, Ayre D, Whelan R, Young A (2011). Patterns of hybridization and asymmetrical gene flow in hybrid zones of the rare *Eucalyptus aggregata* and common *E. rubida*. Heredity.

[47] Wallace LE, Culley TM, Weller SG, Sakai AK, Kuenzi A, Roy T, Wagner WL, Nepokroeff M (2011). Asymmetrical gene flow in a hybrid zone of *Hawaiian Schiedea* (Caryophyllaceae) species with contrasting mating systems. PLoS One.

[48] Godbout J, Yeh FC, Bousquet J (2012). Large-scale asymmetric introgression of cytoplasmic DNA reveals Holocene range displacement in a north American boreal pine complex. Ecol Evol.

[49] Li Z, Zou J, Mao K, Lin K, Li H, Liu J, Källman T, Lascoux M (2011). Population genetic evidence for complex evolutionary histories of four high altitude juniper species in the Qinghai–Tibetan plateau. Evolution.

[50] Lepais O, Petit R, Guichoux E, Lavabre J, Alberto F, Kremer A, Gerber S (2009). Species relative abundance and direction of introgression in oaks. Mol Ecol.

[51] Adams RP (2013). Hybridization between *Juniperus grandis*, *J. occidentalis* and *J. osteosperma* in Northwest Nevada I: Terpenes, leviathan mine, Nevada. Phytologia.

[52] Adams RP (2013). Hybridization between *Juniperus grandis, J. occidentalis* and *J. osteosperma* in Northwest Nevada II: Terpenes, Buffalo Hills, northwestern Nevada. Phytologia.

[53] Adams RP, Kistler J (1991). Hybridization between *Juniperus erythrocarpa* Cory and *Juniperus pinchotii* Sudworth in the Chisos mountains, Texas. Southwest Natl.

[54] Adams RP, Johnson S, Coombes AJ, Caamaño L, González-Elizondo MS (2018). Preliminary examination of hybridization and introgression between *Juniperus flaccida* and *J. poblana*: nrDNA and cpDNA sequence data. Phytologia.

[55] Levin DA (2002). The role of chromosomal change in plant evolution.

[56] Petit C, Bretagnolle F, Felber F (1999). Evolutionary consequences of diploid–polyploid hybrid zones in wild species. Trends Ecol Evol.

[57] Husband BC, Sabara HA (2004). Reproductive isolation between autotetraploids and their diploid progenitors in fireweed, *Chamerion angustifolium* (Onagraceae). New Phytol.

[58] Montesinos D, García-Fayos P, Verdú M (2012). Masting uncoupling: mast seeding does not follow all mast flowering episodes in a dioecious juniper tree. Oikos.

[59] Montesinos D, De Luís M, Verdu M, Raventós J, García-Fayos P (2006). When, how and how much: gender-specific resource-use strategies in the dioecious tree *Juniperus thurifera*. Ann Bot.

[60] Rodriguez-García E, Olano JM, Leroux O, Mezquida ET (2019). Deciphering the role of reproductive investment, pollination success and predispersal seed predation on reproductive output in *Juniperus thurifera*. Plant Ecol Divers.

[61] Amaral-Franco J, Juniperus L, Castroviejo S, Laínz M, López González G, Monserrat P, Muñoz Garmendia F, Paiva J, Villar L (1986). In Flora Ibérica. Plantas Vasculares de la Península Ibérica e Islas Baleares.

[62] Pérez-Latorre AV, Cabezudo B, Juniperus L, Blanca G, Cabezudo B, Cueto M, Morales CT, Salazar C (2011). Flora Vascular de Andalucía Oriental.

[63] Junta de Andalucía. 2019. Agroclimatic stations data. Agricultural and fisheries research and training institute. Counseling of agriculture, livestock, fisheries and sustainable development. (https://www.juntadeandalucia.es/agriculturaypesca/ifapa/ria/servlet/FrontController), accessed 2019.

[64] Levetin E (1998). A long-term study of winter and early spring tree pollen in the Tulsa, Oklahoma atmosphere. Aerobiologia.

[65] Frenguelli G, Spieksma FTM, Bricchi E, Romano B, Mincigrucci G, Nikkels A, Dankaart W, Ferranti F (1991). The influence of air temperature on the starting dates of the pollen season of *Alnus* and *Populus*. Grana.

[66] Boyer WD (1973). Air temperature, heat sums, and pollen shedding phenology of longleaf pine. Ecology.

[67] Mohanty RP, Buchheim MA, Anderson J, Levetin E (2017). Molecular analysis confirms the long-distance transport of *Juniperus ashei* pollen. PLoS One.

[68] Szczepanek K, Myszkowska D, Worobiec E, Piotrowicz K, Ziemianin M, Bielec-Bąkowska Z (2017). The long-range transport of Pinaceae pollen: an example in Kraków (southern Poland). Aerobiologia.

[69] Neale DB, Wheeler NC (2019). The conifers. The conifers: genomes, variation and evolution.

[70] Stewart JF, Tauer CG, Nelson C (2012). Bidirectional introgression between loblolly pine (*Pinus taeda* L.) and shortleaf pine (*P. echinata* Mill.) has increased since the 1950s. Tree Genet Genom.

[71] Sarvas R (1962). Investigations on the flowering and seed crop of *Pinus silvestris*. Comm Instituti Forestalis Fenniae.

[72] Koski V (1970). A study of pollen dispersal as a mechanism of gene flow in conifers. Comm Instituti Forestalis Fenniae.

[73] Nichols H, Kelly P, Andrews J (1978). Holocene palaeo-wind evidence from palynology in Baffin Island. Nature.

[74] Campbell ID, McDonald K, Flannigan MD, Kringayark J (1999). Long-distance transport of pollen into the Arctic. Nature.

[75] Lindgren D, Paule L, Xihuan S, Yazdani R, Segerström U, Wallin J-E, Lejdebro ML (1995). Can viable pollen carry scots pine genes over long distances?. Grana.

[76] Varis S, Pakkanen A, Galofré A, Pulkkinen P (2009). The extent of south-north pollen transfer in Finnish scots pine. Silva Fenn.

[77] Williams CG (2010). Long-distance pine pollen still germinates after meso-scale dispersal. Am J Bot.

[78] Caramiello R, Potenza A, Siniscalco C (1990). Relationship between distribution of *Juniperus communis* L. ssp. *communis* in western Alps, its pollen morphology and viability. Allionia.

[79] Robledo-Arnuncio JJ (2011). Wind pollination over mesoscale distances: an investigation with scots pine. New Phytol.

[80] Robledo-Arnuncio J, Gil L (2005). Patterns of pollen dispersal in a small population of *Pinus sylvestris* L. revealed by total-exclusion paternity analysis. Heredity.

[81] Junta de Andalucía. 2019. Threatened Flora system of Andalusia (FAME). Accessed 2019.

[82] Taylor SA, Larson EL, Harrison RG (2015). Hybrid zones: windows on climate change. Trends Ecol Evol.

[83] Hamilton JA, Amanda R, Aitken SN (2015). Fine-scale environmental variation contributes to introgression in a three-species spruce hybrid complex. Tree Genet Genomes.

[84] Sora D, Kron P, Husband B (2016). Genetic and environmental determinants of unreduced gamete production in *Brassica napus*, *Sinapis arvensis* and their hybrids. Heredity.

[85] Wang J, Li D, Shang F, Kang X (2017). High temperature-induced production of unreduced pollen and its cytological effects in *Populus*. Sci Rep.

[86] Romo A, Hidalgo O, Boratyński A, Sobierajska K, Jasińska AK, Vallès J, Garnatje T (2013). Genome size and ploidy levels in highly fragmented habitats: the case of western Mediterranean *Juniperus* (Cupressaceae) with special emphasis on *J. thurifera* L. Tree Genet Genomes.

[87] Peña J, Cuadrat J, Sánchez M (2002). El Clima de la Provincia de Teruel. Cartillas Turolenses, no 20.

[88] Aupí V. El triángulo de hielo (Teruel – Calamocha – Molina de Aragón). Estudio climático del Polo del Frío español. Teruel: Dobleuve Comunicación; 2014.

[89] Fitchett JM, Grab SW, Thompson DI (2015). Plant phenology and climate change: Progress in methodological approaches and application. Prog Phys Geogr.

[90] Wadgymar SM, Ogilvie JE, Inouye DW, Weis AE, Anderson JT (2018). Phenological responses to multiple environmental drivers under climate change: insights from a long-term observational study and a manipulative field experiment. New Phytol.

[91] Adams RP, Bartel J, Price R (2009). A new genus, *Hesperocyparis*, for the cypresses of the Western hemisphere (Cupressaceae). Phytologia.

[92] Adams RP, Kauffmann ME (2010). Variation in nrDNA, and cpDNA of *Juniperus californica*, *J. grandis*, *J. occidentalis* and *J. osteosperma* (Cupressaceae). Phytologia.

[93] Adams RP (1975). Statistical character weighting and similarity stability. Brittonia.

[94] Garnatje T, Vallès J, Garcia S, Hidalgo O, Sanz M, Canela MÁ, Siljak-Yakovlev S (2004). Genome size in *Echinops* L. and related genera (Asteraceae, Cardueae): karyological, ecological and phylogenetic implications. Biol Cell.

[95] Bourge M, Brown SC, Siljak-Yakovlev S (2018). Flow cytometry as tool in plant sciences, with emphasis on genome size and ploidy level assessment. Genet Appl.

