# Supplementary material for: Gene flow between diploid and tetraploid junipers - two contrasting evolutionary pathways in two Juniperus populations
Source: BMC Evol Biol. 2020 Nov 9;20:148. doi: 10.1186/s12862-020-01688-3 (PMC7650182; doi:10.1186/s12862-020-01688-3)
Supplement: Supplementary file 2 — Additional file 2. Collection information and field notes. [file 12862_2020_1688_MOESM2_ESM.docx]

Additional file 2:

**Title**: Collection information and field notes

**Description:** we are providing specific details for our collected samples such as the location name, address and GPS codes.

**Eastern Iberian Range**

1. **Abejuela**, Sierra de Javalambre, Teruel county, Aragón region, Spain.

***Juniperus thurifera* L. var. *thurifera****,* trees to 4-5 m, common on limestone with *J. sabina* and a few putative hybrids. Near Municipality Abejuela, Teruel County, 39° 56' 28" N, 0° 52' 51" W. 1414 m, 1 June 2019, Spain, Coll. *Robert P. Adams 15616-15625*, with Carlos Fabregat, Silvia Lopez Udias.

***Juniperus sabina* L. var. *sabina*** (cp determination by cpDNA trnS-trnG sequence), prostrate shrubs (0.2 m high x 3 to 5 m wide) to very decumbent shrubs (0.5 m x 3 to 5 m wide), common on limestone with *J. thurifera* and a few putative hybrids. Near Municipality Abejuela, Teruel County, 39° 56' 28" N, 0° 52' 51" W. 1414 m, 1 June 2019, Spain, Coll. *Robert P. Adams* *15626-15645*, with Carlos Fabregat, Silvia Lopez Udias*.*

***Juniperus x cerropastorensis*** JM Aparicio & PM Uribe-Echebarria, putative *Juniperus sabina x J. thurifera* hybrids, spreading shrubs (0.5 - 2 m high x 3 to 5 m wide) with spreading to upright tipped limbs. Intermediate in morphology between *J.* *sabina* and *J.* *thurifera*, scattered (12-15 seen) on limestone with *J. sabina* and *J. thurifera*. Near Municipality Abejuela, Teruel County, 39° 56' 28" N, 0° 52' 51" W. 1414 m, 1 June 2019, Spain, Coll. *Robert P. Adams 15646-15654,* with Carlos Fabregat, Silvia Lopez Udias. *Juniperus x cerropastorensis*, spreading shrub (4 m high x 6 m wide) with spreading to upright tipped limbs. Intermediate in morphology between *J.* *sabina* and *J.* *thurifera*, present on limestone with *J. sabina* and *J. thurifera*. Near Municipality Abejuela, Teruel County, 39° 56' 33" N, 0° 52' 49" W. 1504 m, 1 June 2019, Spain, Coll. *Robert P. Adams 15655*, with Carlos Fabregat, Silvia Lopez Udias. *Juniperus x cerropastorensis,* spreading shrubs (0.5 - 2 m high x 3 to 5 m wide) with spreading to upright tipped limbs. Intermediate in morphology between *J.* *sabina* and *J.* *thurifera*. Scattered (3-5 seen) on limestone with *J. sabina* and *J. thurifera*. Near Municipality Abejuela, Teruel County, 39° 56' 33" N, 0° 52' 49" W. 1504 m, June 2019, Spain, Coll. *Robert P. Adams 15656-15658* with Carlos Fabregat, Silvia Lopez Udias.

1. **Bronchales**, Sierra de Albarracín, Teruel County, Aragón region, Spain.

***Juniperus x cerropastorensis***, from mixed population of *J. thurifera* and *J. sabina* with *J. communis* ssp. *hemispherica*, 6 putative hybrid plants sampled. Near Bronchales, Albarracin Mountains, Teruel County, Aragon, 40° 31' 37.56" N, 1° 31' 25.68" W. 1350 m. 26 July 2019, Spain, Coll. *Carlos Fabregat & Silvia Lopez-Udias, SxT BRO 1-6*. Lab accession *Robert P. Adams 15766, 67, 68, 69, 70, 71*,

1. **Puertomingalvo**, Maestrazgo Mountains, Teruel County, Aragón region, Spain.

***Juniperus x cerropastorensis***, from open forest of Scots Pine (*P. sylvestris*) with *J. sabina* and *J. communis* ssp. *hemispherica*. *J. thurifera* and *J. phoenicea*, ca 5 km away. Maestrazgo Mountains. Teruel County, Aragon. 40° 19' 57.72" N, 0° 28' 23.52" W. 1470 m. 17 August 2019 Spain, Coll. Carlos Fabregat & Silvia Lopez-Udias, *SxT PUE 1-3*. Lab accession *Robert P. Adams 15772, 15773, 15774*.

**Sierra de Baza: Baza, Granada, Spain**,

***Juniperus sabina* L. var. *sabina*** and/or ***J. sabina.* var. *balkanensis*** RP Adams & AN Tashev,

**High elevation site (2188 m)**, prostrate shrubs (0.2 m high x 3 to 5 m wide) to very decumbent shrubs (0.5 m x 3 to 5 m wide), common on limestone with *J. communis.* Cerro de Baza, Collado de la Mina, Granada province, high elevation site 1. 37° 22' 57" N, 02° 49' 59" W. 2188 m. 4 June 2019, Spain, Coll. *Robert P. Adams 15662-15676*(BZsab1-15), with Carlos Salazar Mendias, Joaquín Altarejos.

**Middle** **elevation site (2024 m),** prostrate shrubs (0.2 m high x 3 to 5 m wide) to very decumbent shrubs (0.5 m x 3 to 5 m wide), common on limestone with *J. communis.* Sierra de Baza, near Prados del Rey, Granada province, medium elevation site 2. 37° 22' 28" N, 02° 51' 03" W. 2024 m. 4 June 2019, Spain, Coll. *Robert P. Adams 15677-15691* (BZsab16-30), with Carlos Salazar Mendias, Joaquín Altarejos.

Voucher specimens for all collections are deposited at Baylor University Herbarium (BAYLU).
